# Robustness assessment of Muscat coastal highway network (CHN) under multi-hazard scenarios focusing on traffic stability and adaptation measures

**DOI:** 10.1038/s41598-024-79730-3

**Published:** 2024-12-24

**Authors:** Abdullah Ansari, Issa El-Hussain, Yousuf Al Shijbi, Pranjal Mandhaniya, Ayed E. Alluqmani, Khalifa Al-Jabri

**Affiliations:** 1 Earthquake Monitoring Center, Sultan Qaboos University, PC: 123 Al Khoudh, Muscat, Oman; 2 Department of Mechanical and Industrial Engineering, Norwegian University of Science and Technology Trondheim, Trondheim, Norway; 3 Department of Civil Engineering, Faculty of Engineering, Islamic University of Madinah, Al-Madinah Al-Munawarah, Saudi Arabia; 4 Department of Civil and Architectural Engineering, Sultan Qaboos University, PC: 123 Al Khoudh, Muscat, Oman

**Keywords:** Muscat, Coastal highway, Traffic, Economic loss, Reliability, Resilience, Natural hazards, Engineering

## Abstract

This study critically examines the reliability and resilience of the Muscat coastal highway network (CHN) under the compounded effects of earthquakes and floods, representing interacting multi-hazard scenarios. The analysis utilized fragility functions for both earthquake-induced and flood-induced landslides, integrating these with traffic data for selected highway links to estimate bridge damage and assess CHN functionality in post-hazard conditions. Economic sensitivity analysis revealed a significant increase in costs due to flood-induced landslides, emphasizing the impact of dominant intensity measures on network costs and traffic flow. The analysis categorized Muscat areas into low, moderate, and high resilience based on hazard susceptibility and infrastructure quality, revealing that over 50% of highway links require retrofitting, highlighting the need for enhanced flood management and infrastructure improvements. The resilience assessment highlighted the necessity for targeted retrofitting to mitigate damage and reduce economic losses, particularly for highway links with bridges of high failure probabilities that face prolonged recovery times. The results provide valuable insights for designers, consultants, policymakers, and decision-makers in developing effective post-hazard mitigation strategies for Muscat and similar coastal cities.

## Introduction

Naturally occurring hazards such as earthquakes and floods, pose severe risks to transportation infrastructure, particularly in tectonically active coastal regions. These hazards can cause significant damage to roadbeds, bridges, overpasses, and tunnels, leading to road closures, accidents, and costly repairs. Coastal highway networks (CHN) are especially vulnerable, as earthquakes can trigger landslides and tsunamis^1–5^, while floods can exacerbate damage through erosion and debris. Oman has historically faced numerous natural disasters, such as earthquakes, floods, and cyclones, which have resulted in both economic and structural losses depending on the hazard’s intensity^6,7^. Fragility analysis is commonly applied to assess bridge performance, identifying critical vulnerability zones^8,9^. Previous studies across the United States, United Kingdom, Germany, Chile, South Korea, and other countries have focused on reliability assessments of coastal highways^10–16^. Multi-hazard impacts lead to economic losses^17,18^, driving retrofitting measures and structural safety improvements^19,20^. D’Amato et al.^21^ and Laguardia et al.^22^ conducted damage assessments, proposing loss curves and Expected Annual Loss (EAL) models using 2009 L’Aquila earthquake data to improve seismic risk assessment. Similar fragility-based damage assessments were performed for the 2012 Emilia earthquake^23^.

Given Muscat’s coastal location, future seismic activities could trigger secondary hazards like landslides, tsunamis, and liquefaction, potentially causing severe damage to transportation infrastructure, including tunnels and bridges. Historically, floods and cyclones have significantly impacted Muscat’s coastal highways. For instance, during Cyclone Gonu in 2007, several bridges, such as the Al Hail Bridge, experienced foundation settlement due to extreme floodwaters, leading to substantial disruptions in traffic flow^24^. Similarly, in 2010, Cyclone Phet caused extensive damage to Sultan Qaboos Highway, including erosion undermining bridge foundations^6^ and resulting in major traffic disruptions. Flash floods have also led to landslides and road blockages in areas with high flood depths, further compounding traffic flow issues. Notably, the 2010 floods caused significant landslides in the areas surrounding the Al Amerat region, where heavy rainfall resulted in saturated soils, leading to soil instability and road blockages^25^.

Floods can significantly contribute to flood-induced landslides, especially in areas with steep terrain or loose soil. Intense rainfall associated with floods increases the water content in the soil, reducing its shear strength and making it more susceptible to sliding. As the soil becomes saturated, it loses its ability to hold onto slopes, leading to instability and potential landslides^26,27^. In Muscat, such flood-induced landslides can obstruct roads and damage transportation networks, further compounding the impact of the floods. The Earthquake Monitoring Center (EMC) at Sultan Qaboos University (SQU) has previously mapped seismic hazards^28,29^ in the Muscat region and identified liquefaction potential and local site effects^30,31^. Previous studies have identified flood vulnerable zones^32,33^ and emphasized the need for detailed analysis,^34,35^ but a comprehensive study on the resilience and reliability of transportation infrastructure has been lacking. Given these historical impacts and the potential for future multi-hazard scenarios, it is essential to conduct robustness checks on coastal highways.

The present study assessed the reliability and resilience of Muscat CHN by visualizing damage impacts and developing functionality maps under selected multi-hazard scenarios. These assessments are vital for understanding how transportation infrastructure withstands simultaneous or sequential impacts from earthquakes, floods, and secondary hazards. Aligned with Oman Vision 2040, the study focused on enhancing adaptation measures for coastal highways, addressing economic sensitivity to structural losses, and identifying vulnerable elements. In addition to this, it is providing scalable methodologies that can strengthen national infrastructure planning and policy development, promoting sustainable urban growth. The dataset can be adapted to other coastal cities with similar hazards and applied globally for infrastructure case studies with different hazard profiles, contributing to United Nation’s Sustainable Development Goals (SDGs) 9 and 11 for innovative infrastructure and sustainable cities.

## Project detailing and methodology

**Fig. 1:**
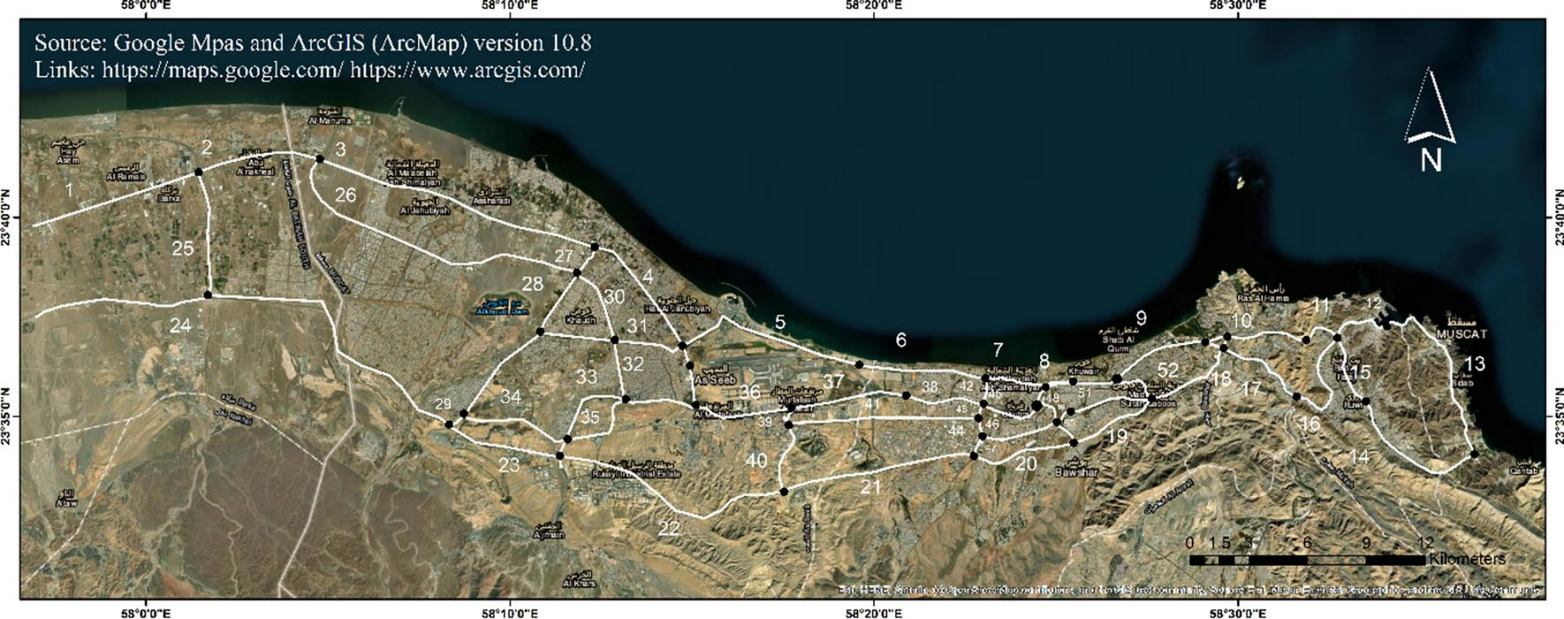
Study area map showing highway links and bridges considered. Bridges are marked with black dots and highway links with white lines. The base map is quoted from the publicly available Google satellite imagery maps (https://maps.google.com/). The figure is created using ArcGIS (ArcMap) (v.10.8, https://www.arcgis.com/).

The coastal highway network (CHN) of Muscat is crucial for linking key areas, including the city center, Port Sultan Qaboos, and Muscat International Airport. The Sultan Qaboos Highway is a major route facilitating smooth transit across the city. Important bridges such as the Al Khuwair and Ruwi bridges enhance traffic flow and connectivity between urban centers. In this study, the focus is on the coastal highway network extending from Mutrah to Barka, featuring 38 bridges and 52 highway links, detailed in Fig. 1 and Appendix I. Major congestion points are found in Bawshar, Al Khuwair, Ghubra, and Madinat Al Sultan Qaboos. The longest bridges, including Al Khoudh Bridge (KHB), Al Mabelah Bridge (AMB), and Mawaleh Bridge (MWB) in Seeb, connect old Muscat with newly developed areas in Seeb and Barka. Ruwi MBD Bridge (RMB) and Amerat Bridge (MRB) link the eastern and western ends of Muscat, connecting areas like Qurm, Azaiba, and Al Khoud.

**Fig. 2:**
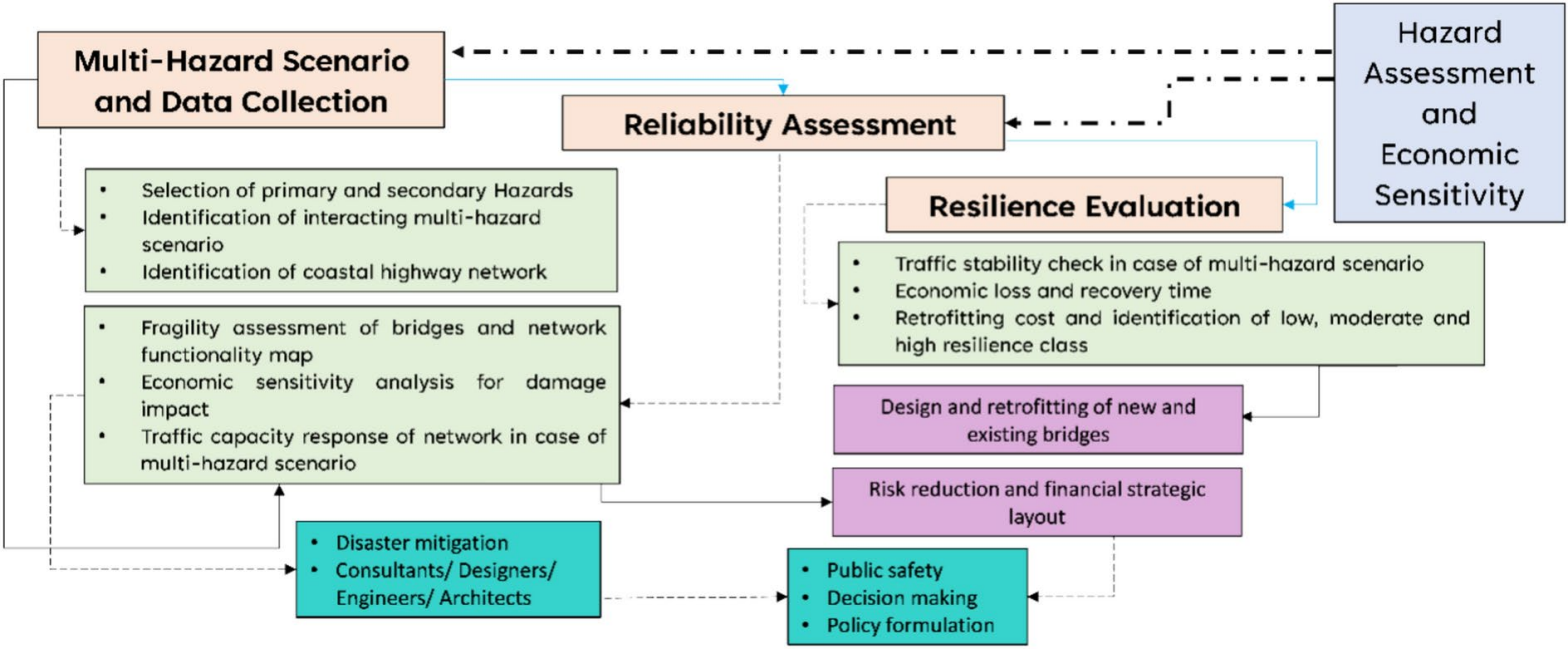
Flowchart detailing the steps for present study goals and outcomes.

Earthquakes and floods are identified as primary hazards in Muscat, with secondary hazards including induced landslides and liquefaction in this study. Multi-hazard scenarios are analyzed by integrating combinations of these primary and secondary hazards. Detailed hazard combinations are presented in Table 1. To assess network functionality, the initial step involves hazard identification. For seismic assessment, the seismic potential data for Muscat from OSC ^36^ is utilized, with return periods (RPs) of 475 and 2475 years^28^. Flood hazard input is derived using the classification map proposed by^33^, which categorizes susceptibility from very low to very high. The overall procedure, from data collection to reliability and resilience assessment, is illustrated in Fig. 2. Here, the robustness of the coastal highway network is defined by combining reliability assessment and resilience evaluation. Reliability is measured in terms of economic loss resulting from bridge damage, while resilience is evaluated based on traffic stability and the retrofitting costs associated with repairing damaged bridges. This integrated approach provides a comprehensive framework to assess the network’s capacity to withstand, recover, and adapt to adverse events with minimal economic and operational impacts.

**Table 1:** Details of the selected hazard scenarios for the present study

Primary hazard			Secondary hazard			Multi-Hazard		
P1	Earthquake	EQ	S1	Earthquake-induced landslide	EQLD	M1	P1+P2	= EQ+FD
			S2	Earthquake-induced liquefaction	EQLN	M2	S1+S2	= EQLD+ EQLN
P2	Flood	FD	S3	Flood induced landslide	FDLD	M3	S1+P2	= EQLD+FD
						M4	S1+S3	= EQLD+FDLD
						M5	S2+P2	= EQLN+FD
						M6	S2+S3	= EQLN+FDLD

To evaluate the probability of various damage states in Muscat CHN, fragility functions^37,38^ are constructed using selected dominant intensity measures (${IM}_{D}$) for each hazard. To effectively address the intricate interactions of multi-hazard effects, empirical relationship developed by FEMA^39^ for fragility analysis were referred in this study. These functions are used to integrate damage probabilities from combined hazards, illustrating the cumulative impact of earthquakes and floods, as shown in Eqs. (1) and (2). In this study, only bridges within the Muscat CHN whose structural design aligns with the specifications outlined in FEMA^39^ are considered, ensuring consistency in the results.

1 $$P\left[ {DS = DS_{i} } \right] = P\left[ {\left\{ {\left. {DS \ge DS_{i} } \right|P1} \right\} \cup \left\{ {\left. {DS \ge DS_{i} } \right|P2} \right\}} \right] - P\left[ {\left\{ {\left. {DS \ge DS_{i + 1} } \right|P1} \right\} \cup \left\{ {\left. {DS \ge DS_{i + 1} } \right|P2} \right\}} \right]\;1 \le i \le n$$

2 $$P\left[ {DS = DS_{i} } \right]{ = }P\left[ {\left\{ {\left. {DS \ge DS_{i} } \right|P1} \right\} \cup \left\{ {\left. {DS \ge DS_{i} } \right|P2} \right\}} \right]\;{\text{i }} = {\text{n}}$$

These two equations, in a simplified form, can be defined as:

3 $$P[\{DS\ge {DS}_{i}\mid P1\}\cup \{DS\ge {DS}_{i}\mid P2\}] = P[DS\ge {DS}_{i}\mid P1] +P[DS\ge {DS}_{i}\mid P2] -P[DS\ge {DS}_{i}\mid P1] \times P[DS\ge {DS}_{i}\mid P2]$$

In the equations mentioned earlier, ${DS}_{i}$ represents the ${i}^{th}$ damage state linked to the chosen hazard scenario impacting Muscat CHN. The values of P1 and P2 can be substituted using S1, S2, and S3. These calculated damage probabilities serve as inputs for estimating bridge repair costs (${\mu }_{RCR}$) and highway traffic capacity (${C}_{T}$), following the methodologies outlined in Eqs. (4) and (5) by Nako et al.^40^ and Mehary and Dusicka^41^, respectively.

4 $${\mu }_{RCR}= \sum_{i=2}^{5}{RCR}_{i} P[DS={DS}_{i}\mid {IM}_{D}]$$

5 $${C}_{T}= \sum_{i=1}^{5}\left({P}_{i}\times P[DS= {DS}_{i}\mid {IM}_{D}]\right)\le 1.0$$

In the preceding equations, ${\mu }_{RCR}$ and ${P}_{i}$ denote the mean repair cost ratio *(*$RCR$*)* and the probability percentage of the *i*th damage state, respectively. For an in-depth evaluation of the functionality of highway links within Muscat CHN, the link damage index ($LDI$) is computed using Eqs. (6) and (7) to account for varying levels of structural damage. The bridge damage index ($BDI$) spans a continuum from 0 to 1.0, where a $BDI$ of 0 signifies an undamaged structure, and a $BDI$ of 1.0 indicates a total structural collapse. Analyzing the collective $LDI$ values across all specified links enables the characterization of the operational integrity post-hazard scenario, providing a quantitative assessment of network reliability.

6 $$LDI= \left\{\begin{array}{c}\sqrt{{\sum }_{i=1}^{52}{\left[BDI(i)\right]}^{2}} BDI(i)<1\\ \infty BDI\left(i\right)=1\end{array}\right.$$

The relationship between $BDI$ and the failure probability of each bridge is captured in how $BDI$ reflects the extent of damage. $BDI(i)$ close to 1 suggests a higher probability of failure, as the bridge approaches full damage, while values significantly less than 1 indicate lower failure probability. By aggregating $BDIs$, the $LDI$ represents the overall damage scenario within the network, emphasizing the criticality of highly damaged bridges in influencing the probability of network failure. The operational capability of CHN can be significantly affected by the structural damage states and the hazard intensity. This influence is quantified through the travel time and connectivity reliability ($TTCR$) curve, which is plotted using data corresponding to various RPs across selected hazard scenarios. Calculating $TTCR$ requires summing two primary components: connectivity reliability ($CR$) and travel time reliability ($TTR$), as detailed in Eq. (7) for 52 selected links for Muscat. In this equation, ​${f}_{52}$ denotes the connectivity measure between the origin and destination points (O-D) for distinct highway links, offering a statistical evaluation of the network’s connectivity efficiency. The variables ${t}_{bj}$ and ${t}_{aj}$ represent the shortest travel time from the origin to the destination before and after an earthquake, respectively. By assessing these metrics, the $TTCR$ curve provides a comprehensive understanding of the coastal highway network’s resilience and operational performance under multi-hazard scenarios.

7 $$TTCR = \frac{\left(CR\right)+\left({TTR}_{j}\right)}{2} = \frac{\left(\frac{{\sum }_{1}^{52}{f}_{52}({y}_{1}, {y}_{2}, ...{y}_{52})}{52}\right)+\left(\frac{{t}_{bj}}{{t}_{aj}}\right)}{2}$$

In this study, the origin and destination points for each travel are typically selected based on major traffic hubs such as commercial centers, residential districts (e.g., Seeb or Mutrah), and key junctions like those connecting to the airport or industrial areas. These points are critical for evaluating the CHN performance during daily operations and hazard events. To account for the effects of damage to each bridge on all travels within the highway network, the model evaluated each of the 52 links in terms of their importance to overall connectivity. The connectivity measure ${f}_{52}\left({y}_{1}, {y}_{2}, ...{y}_{52}\right)$ in Eq. (7) captured the extent to which damage to these bridges impacts the flow of traffic between selected origin–destination pairs. For example, a damaged bridge near the Qurm or the Al Amerat link would significantly disrupt traffic flow, causing delays across the network.

This study conducted a detailed analysis of full recovery time for Muscat CHN by quantifying economic losses from both direct and indirect sources, which is crucial for assessing resilience factors. This approach enables the determination of the total recovery duration required following a disruptive event, incorporating both immediate impacts and subsequent secondary effects^42–45^. The assessment focused on various damage states of bridge structures, evaluated through two primary metrics: expected economic loss (${L}_{e}$) and full recovery time (${t}_{FR}$). The expected economic loss for a specific highway link is derived using the following formula, where ${R}_{D}$ and ${R}_{I}$ represent direct and indirect losses, respectively.

8 $$L_{e} = \left[ {R_{D} } \right] + \left[ {R_{I} } \right] = \left[ {\mathop \sum \limits_{k = 1}^{52} \mathop \sum \limits_{i = 1}^{{m_{b} }} pf_{k,b} \left( {DS = DS_{i} } \right) \times C_{{DS_{i,b} }} } \right]\left[ {R_{n} + R_{t} } \right]$$

As detailed in Eq. (8), *m* ${m}_{b}$ and ${C}_{{DS}_{i,b}}$ denote the damage state and the direct repair costs for the affected bridge, respectively. The term ${R}_{n}$ captures the economic impact related to vehicles navigating detours, which is influenced by the operational costs for cars (${C}_{C}$) and trucks (${C}_{T}$), depending on the condition of the highway link (${l}_{s}$).

9 $${R}_{n}= \sum P({l}_{s})\times \left\{\sum_{i=1}^{{t}_{f}}\left[{C}_{C}\left(1-\frac{{t}_{t}}{100}\right)+{C}_{T}\left(\frac{{t}_{t}}{100}\right)\right]\times \left[ADTD{ \times l}_{d}\right]\right\}$$

10 $${R}_{t}= \sum P({l}_{s})\times \left\{\sum_{i=1}^{{t}_{f}}\left[{C}_{wh}{O}_{C}\left(1-\frac{{t}_{t}}{100}\right)+{C}_{ch} {O}_{T}\left(\frac{{t}_{t}}{100}\right)\right]\times \left[\left(\frac{ADTD{ l}_{d}}{S} \right)+\left(ADTE\times \left(\frac{{l}_{k}}{{S}_{d}}-\frac{{l}_{k}}{{S}_{i}}\right)\right)\right]\right\}$$

In the preceding equations, ${R}_{t}$ represents the monetary value of time losses for users navigating both the detour and the damaged link at a specific damage state. ${t}_{f}$ denotes the time required for all structures to achieve full operational functionality. The parameters include: ${l}_{d}$= Length of the detour, $ADTD$ = Average daily traffic rerouted to the detour, $ADTE$ = Average daily traffic remaining on the damaged link, ${t}_{t}$ = Ratio of average daily truck traffic, ${C}_{wh}$ = Wage rate per hour, ${C}_{ch}$ = Compensation rate per hour, ${O}_{C}$ = Average vehicle occupancy for cars, ${O}_{T}$ = Average vehicle occupancy for trucks, ${l}_{k}$ = Length of the highway link, ${S}_{i}$ = Average speed on the undamaged link, ${S}_{d}$ = Average speed on the damaged link, $S$ = Average speed on the detour

The full recovery time (${t}_{FR}$) is determined by the recovery times associated with various damage states (${t}_{{DS}_{i,b}}$) for each bridge, which can be computed using Eq. (11) as outlined.

11 $${t}_{FR} =\mathit{max}\left\{{T}_{i=1};{T}_{i=2}\dots \dots \dots \dots \dots ;{T}_{i=52}\right\}$$

12 $${T}_{i}= {\sum }_{i=1}^{{m}_{b}}p{f}_{i,b}\left(DS={DS}_{i}\right)\times {t}_{{DS}_{i,b}}$$

In the realm of network analysis, ${t}_{FR}$ represents the recovery period needed for the network to regain its full operational capacity after suffering damage from events like earthquakes or floods^46^. A reduced ${t}_{FR}$ highlights the network’s enhanced resilience ($R$), demonstrating its capability to swiftly bounce back and restore normal functionality.

13 $$R =\frac{1}{{t}_{h}} \underset{{t}_{0}}{\overset{{t}_{0}+{t}_{h}}{\int }}\left(\frac{\left(\frac{1}{P{I}_{t}\times TTT\left(t\right)+P{I}_{d}\times TTD\left(t\right)}\right)-P{I}_{0}}{P{I}_{100}-P{I}_{0}}\right)dt$$

The parameters include: ${t}_{h}$= Time horizon under investigation, $P{I}_{t}$ = Cost-related balancing factor associated with elapsed time, $P{I}_{d}$= Factor related to traveled distance, $TTT\left(t\right)$ = Total travel time, $TTD\left(t\right)$ = Total travel distance, $P{I}_{0}$ = Performance indicator representing no damage, $P{I}_{100}$ = Performance indicator representing complete bridge collapse

In the present study, the maximum resilience (${R}_{max}$) of Muscat CHN is evaluated by analyzing and comparing the resilience of individual highway links (${R}_{i}$), as outlined in Eq. 14. This evaluation informs the strategic prioritization of retrofitting efforts for bridges within the network. Enhancing ${R}_{max}$ through retrofitting is critical for optimizing the network functionality and reducing recovery times.

14 $${R}_{max} =\mathit{max}\left\{{R}_{i=1};{R}_{i=2}\dots \dots \dots \dots \dots ;{R}_{i=52}\right\}$$

## Reliability assessment

In this study, the failure probabilities (${P}_{F}$) of all 38 bridges (B1, B2, …, B38) within the assumed Muscat CHN are rigorously assessed through a comprehensive fragility analysis. This analysis incorporated the dominant intensity measure (${IM}_{D}$) relevant to each hazard scenario. Key bridges, namely WAB, QRB and MRB have been identified as critical under primary hazard conditions, whereas bridges KHB and BSR are prominent under secondary hazard scenarios (Table 2). When evaluating the integrated effects of both primary and secondary hazards, bridges QRB and WTB emerged as the most vulnerable, with respective failure probabilities reaching 0.88. Table 3 delineates the failure probabilities derived from the fragility functions outlined in Eqs. (1) and (2), applicable to both primary (P1 and P1) and secondary (S1, S2 and S3) hazard scenarios. The analysis revealed that the failure probability for P2 and S3, indicative of total structural collapse exceeds 0.7, an alarming threshold that ranks highest among all considered hazard scenarios across various ${IM}_{D}$ classifications.

**Table 2:** Bridge sensitivity analysis

Aspect	Bridge name	Detailing
Selection of fragility curve	Empirical functions given in FEMA^39^	
Critical bridges	Qurm Bridge (QRB)	Concrete girder bridge with reinforced concrete piers and deck slab
	Al Khoud Bridge (KHB)	Pre-stressed concrete box girder with multiple-span continuous deck
	Amerat Bridge (MRB)	Reinforced concrete slab bridge with multi-span piers
	Bousher Foot Bridge (BSR)	Steel box girder with orthotropic steel deck and reinforced concrete piers
Seismic sensitivity (Vulnerable to low-to-moderate PGA < 0.05 g and failure probability for minor damage can be as high as 0.85)	Qurm Bridge (QRB)	Concrete girder bridge with reinforced concrete piers and deck slab
	Wadi Adai Bridge (WAB)	Concrete box girder bridge with steel reinforcement
Flood sensitivity (Flood depths exceeding 4.0 m. and failure probability for minor damage reaches 0.97)	Amerat Bridge (MRB)	Reinforced concrete slab bridge with multi-span piers
	Bousher Foot Bridge (BSR)	Steel box girder with orthotropic steel deck and reinforced concrete piers
Displacement impact (Susceptible to PGD > 3.0 m and failure probability for extensive damage can increase to 0.54)	Al Khoud Bridge (KHB)	Pre-stressed concrete box girder with multiple-span continuous deck
	Qurm Bridge (QRB)	Concrete girder bridge with reinforced concrete piers and deck slab

**Table 3:** Fragility assessment for selected CHN in Muscat

Hazard scenario		Dominating intensity measure (${IM}_{D}$)		Failure probability (${P}_{F}$)			
				Minor (${D}_{1}$)	Moderate (${D}_{2}$)	Extensive (${D}_{3}$)	Collapse (${D}_{4}$)
Primary	P1	Peak ground acceleration (PGA)	< 0.05 g	0.85	0.61	0.23	0.17
			0.05–0.1 g	0.51	0.42	0.18	0.12
			> 0.1 g	0.43	0.21	0.11	0.09
	P2	Flood depth (${D}_{F}$)	< 4.0 m	0.97	0.94	0.82	0.77
			4.0–7.0 m	0.96	0.91	0.56	0.54
			> 7.0 m	0.93	0.87	0.51	0.47
Secondary	S1	Peak ground displacement (PGD)	< 1.0 m	0.87	0.51	0.21	0.13
			1.0–3.0 m	0.73	0.44	0.18	0.09
			> 3.0 m	0.63	0.36	0.14	0.07
	S2	Peak ground displacement (PGD)	< 1.0 m	0.62	0.44	0.11	0.06
			1.0-3.0 m	0.57	0.39	0.11	0.05
			> 3.0 m	0.52	0.37	0.09	0.05
	S3	Peak ground displacement (PGD)	< 1.0 m	0.89	0.85	0.74	0.71
			1.0–3.0 m	0.82	0.79	0.65	0.63
			> 3.0 m	0.77	0.76	0.62	0.54

**Fig. 3:**
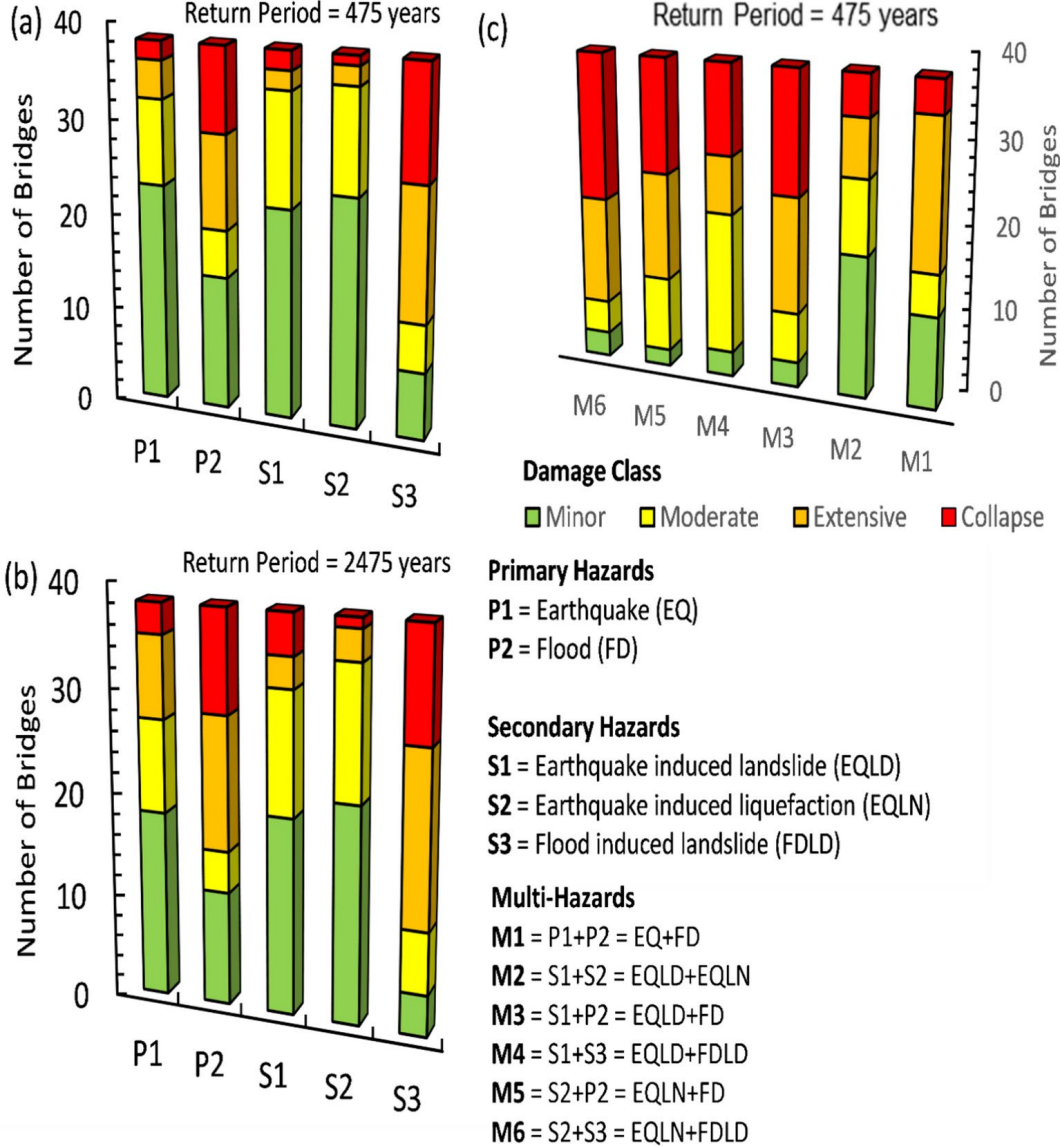
Visualizing damage impact on Muscat bridges from considered hazard scenarios.

Figure 3a demonstrates that for a RP of 475 years, 14 bridges experienced moderate to extensive damage due to earthquake-induced landslides (S1) characterized by a permanent ground displacement (PGD) of 0.93 m. When the RP is extended to 2475 years, over 55% of the bridges exhibited either extensive damage or complete collapse under flood conditions supporting scenarios P2 and S3 (Fig. 3b). This analysis underscored that the severity of the hazard, the geographical positioning of the landslide impact, as well as the structural design and construction quality of the bridges significantly influence the extent and interplay of these damages.

The analysis showed that a total of 28 bridges sustained extensive damage to complete failure under scenario S3, where PGD reached 3.15 m due to flood-induced settlement. This settlement can lead to the subsidence or uneven settling of bridge foundations, compromising vertical alignment and reducing clearance for water flow beneath the structure. Figure 3c demonstrates that scenario M4, resulting from the combined effect of landslides triggered by both seismic and flood events, put 48% of the total bridges in critical conditions marked by extensive damage or total collapse. Additionally, it is notable that scenarios M3 and M6 exhibited a similar damage pattern, despite arising from different combinations of primary and secondary hazards. In these scenarios, bridge segments may become partially or completely submerged, leading to buoyancy issues that could cause lateral displacement or even detachment of segments, posing significant threats to the CHN vulnerability.

**Fig. 4:**
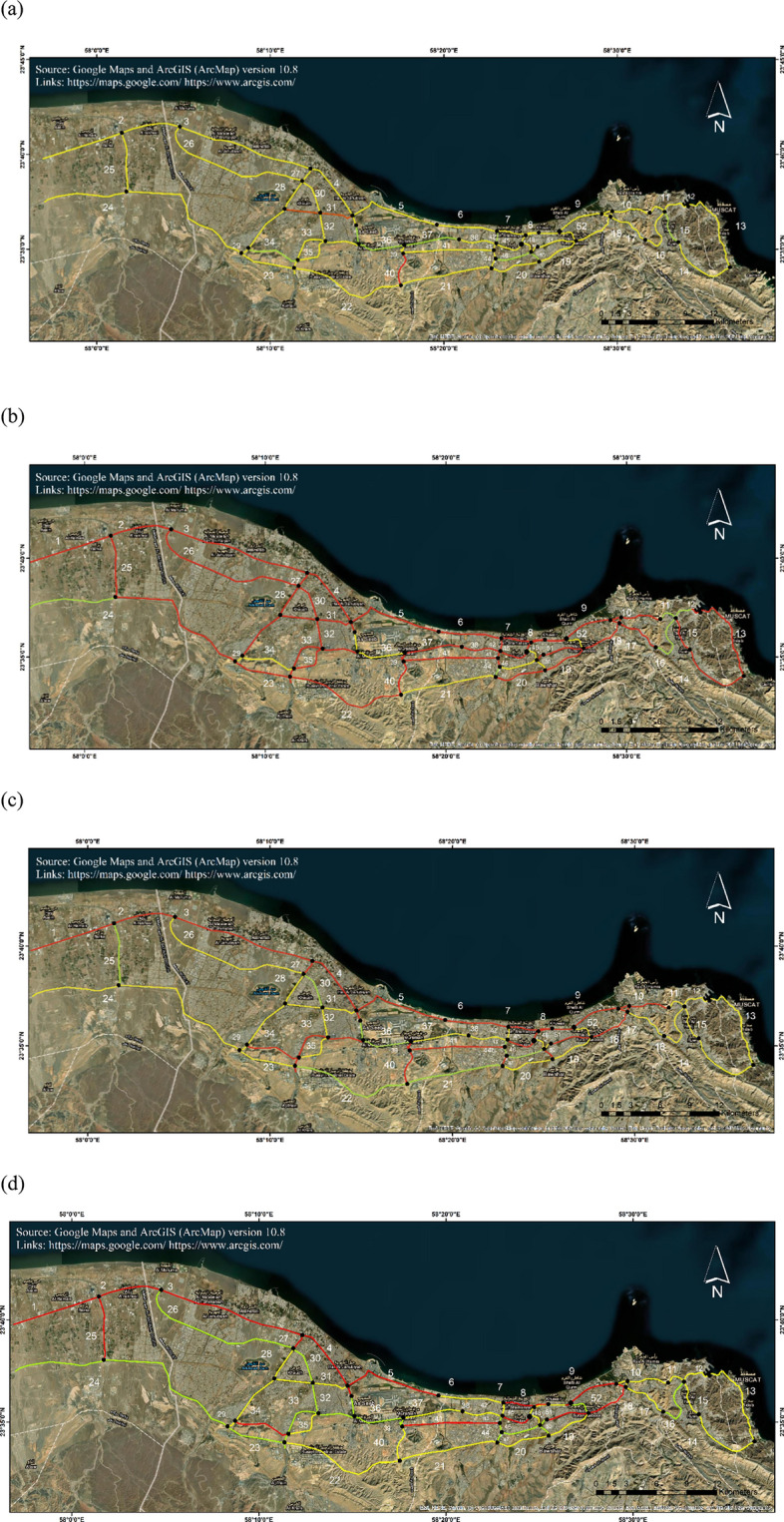
Functionality map of Muscat highways for hazard scenarios: (**a**) M2; (**b**) M3; (**c**) M4 and (**d**) M6. Highway links indicated with red lines denote complete traffic restrictions, while yellow lines signify partial traffic limitations. Green lines represent unrestricted highway links, indicating that the route is safe for vehicular movement in post-disaster scenarios. The base map is quoted from the publicly available Google satellite imagery maps (https://maps.google.com/). The figures are created using ArcGIS (ArcMap) (v.10.8, https://www.arcgis.com/).

The actual traffic capacity of the CHN is meticulously acquired through highway scanning and detailed surveys conducted during fieldwork in Muscat. This empirical data, combined with an in-depth analysis of failure probability under various hazard scenarios were considered for evaluating the overall functionality of Muscat CHN. Figure 4 offers a detailed visualization of the Muscat CHN traffic patterns in specific post-disaster scenarios, designated as M2, M3, M4, and M6. In the M2 scenario, which exclusively considers seismic events, a substantial number of highway links exhibited a likelihood of experiencing partial traffic restrictions. This scenario highlighted the seismic resilience of the CHN, emphasizing the need for targeted seismic retrofitting of key infrastructure elements. The M3, M4, and M6 scenarios incorporate flood events, revealed that highway links in close proximity to coastal regions, particularly links 4–13, 17, 30, 42–47, and 52, are markedly susceptible to structural damage. These links may face a significant risk of complete traffic restrictions, presenting substantial challenges to post-disaster transportation logistics. Flood-induced hydrodynamic forces are likely to exacerbate the deterioration of these infrastructures, accelerating corrosion of bridge components and potentially leading to structural failure. This underscored the critical need for robust hydrological and structural mitigation strategies to preserve functionality.

Conversely, highway links 22, 25, and 36 exhibited no significant issues concerning traffic restrictions under any of the hazard scenarios. Their ability to consistently maintain seamless traffic flow highlighted their robust design and strategic importance in ensuring network connectivity. $LDI$ analysis revealed that highway links 43–46 have reached a critical $LDI$ threshold of 1.00, indicating imminent threats to their structural integrity and operational functionality (Table 4). Additionally, links 5, 15, 27, 28, 49, and 50 also exhibited an $LDI$ of 1.00, marking them as highly vulnerable to multiple hazards. These links are critical nodes within the Muscat CHN, and any compromise in their structural integrity could result in severe traffic disruptions, especially in areas adjacent to the epicenter of an earthquake or coastal flood zone. Proactive maintenance and resilience-enhancing measures are essential to safeguard these vulnerable nodes against multi-hazard impacts.

**Table 4:** Link damage index ($LDI$) under chosen hazard scenarios

Link number	Multi-hazard scenario					
	M1	M2	M3	M4	M5	M6
1	0.69	0.24	0.51	0.47	0.45	0.41
2	0.77	0.28	0.65	0.60	0.55	0.50
3	0.69	0.26	0.62	0.57	0.56	0.51
4	0.77	0.26	0.71	0.65	0.67	0.61
5	1.00	0.29	1.00	1.00	1.00	1.00
6	0.94	0.19	0.88	0.80	0.97	0.89
7	0.51	0.32	0.50	0.46	0.54	0.50
8	0.54	0.29	0.54	0.50	0.47	0.43
9	0.63	0.24	0.60	0.56	0.54	0.50
10	0.67	0.31	0.62	0.57	0.61	0.56
11	1.00	0.28	0.95	0.87	0.89	0.81
12	1.00	0.32	1.00	1.00	1.00	1.00
13	1.00	0.31	1.00	1.00	1.00	1.00
14	1.00	0.30	1.00	1.00	1.00	1.00
15	1.00	0.30	1.00	1.00	1.00	0.98
16	0.57	0.33	0.53	0.50	0.50	0.47
17	0.87	0.25	0.74	0.69	0.63	0.57
18	0.73	0.30	0.65	0.60	0.57	0.52
19	0.59	0.33	0.55	0.51	0.50	0.46
20	1.00	0.30	0.97	0.89	0.89	0.81
21	0.79	0.28	0.65	0.60	0.53	0.49
22	1.00	0.38	1.00	0.96	1.00	0.95
23	0.78	0.32	0.76	0.71	0.68	0.62
24	1.00	0.31	0.94	0.87	0.85	0.78
25	1.00	0.32	0.99	0.92	0.90	0.82
26	1.00	0.36	1.00	0.96	0.98	0.90
27	1.00	0.31	1.00	1.00	0.98	0.89
28	1.00	0.32	1.00	1.00	1.00	0.94
29	1.00	0.25	1.00	0.99	1.00	1.00
30	0.59	0.23	0.60	0.55	0.55	0.50
31	0.53	0.22	0.49	0.46	0.43	0.40
32	1.00	0.40	1.00	1.00	1.00	0.98
33	0.46	0.37	0.56	0.53	0.51	0.48
34	0.82	0.25	0.71	0.65	0.72	0.66
35	0.93	0.32	0.81	0.75	0.73	0.67
36	0.75	0.35	0.85	0.78	0.78	0.71
37	0.75	0.36	0.88	0.82	0.80	0.73
38	0.91	0.35	0.92	0.85	0.81	0.74
39	0.82	0.35	0.95	0.88	0.82	0.75
40	0.83	0.35	0.99	0.92	0.83	0.75
41	0.99	0.42	1.00	0.95	0.91	0.83
42	0.90	0.39	1.00	0.98	0.90	0.82
43	0.94	0.46	1.00	1.00	0.98	0.90
44	1.00	0.43	1.00	1.00	0.97	0.88
45	1.00	0.43	1.00	1.00	0.98	0.89
46	1.00	0.41	1.00	1.00	0.98	0.89
47	1.00	0.47	1.00	1.00	1.00	0.96
48	1.00	0.48	1.00	1.00	1.00	0.98
49	1.00	0.53	1.00	1.00	1.00	1.00
50	1.00	0.45	1.00	1.00	1.00	0.98
51	0.82	0.49	1.00	1.00	0.82	0.75
52	0.93	0.48	1.00	1.00	0.85	0.77

**Fig. 5:**
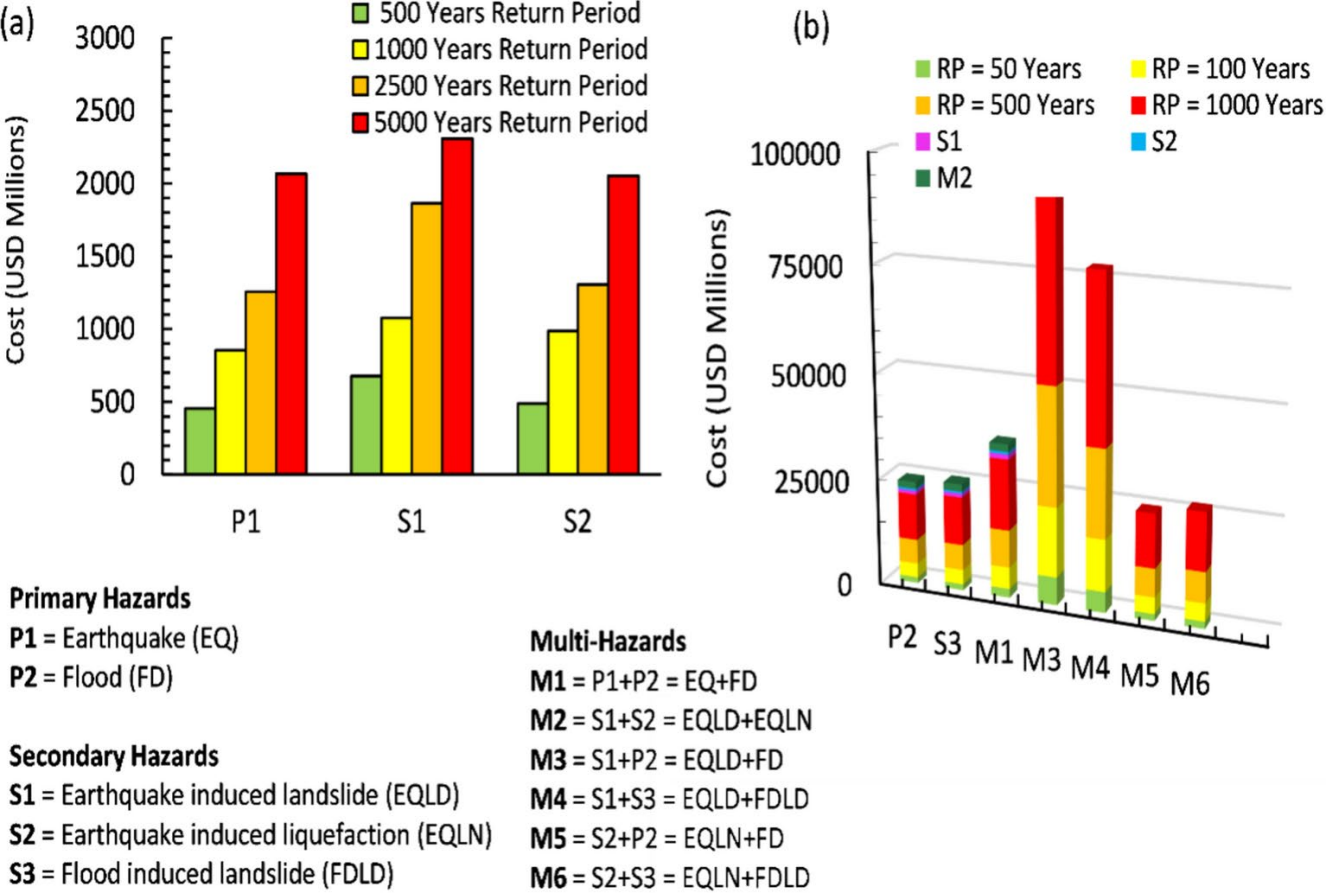
Economic consequences of bridge damage in primary, secondary, and multi-hazard scenarios.

Figure 5a provides a comprehensive analysis of the estimated economic costs, expressed in millions of USD, associated with a spectrum of earthquake-induced hazard scenarios, emphasizing varying RPs. A salient observation emerged at the 1000-year RP, where economic losses from scenarios S1 and S2 converge, exhibiting nearly identical performance. Specifically, Scenario S1 focuses on earthquake-induced landslides, and demonstrated a consistent impact across all earthquake RP cases, thereby highlighting the persistent threat posed by landslides irrespective of the RP. Further, Fig. 5b delves into the compounded impacts of incorporating flood events on loss estimations tied to secondary hazards triggered by earthquakes. Here, the introduction of flood-induced landslide effects alone precipitated a pronounced 126.58% increase in costs compared to isolated flood events, underscoring the exacerbated risks when multi-hazards interact. Conversely, scenarios M5 and M6, which account for liquefaction effects, do not exhibited any significant economic cost increments, suggesting that liquefaction, while impactful, may not contribute markedly to the overall economic losses within these specific scenarios for Muscat CHN. In stark contrast, scenarios M3 and M4 represent the compounded effects of earthquake-induced landslides in conjunction with floods or flood-induced landslides, resulted in the highest economic losses among all assessed multi-hazard scenarios. This combination stands out for its particularly severe adverse economic impacts, rendering it one of the most devastating scenarios analyzed. The analysis underscored that landslide-induced hazards, whether in isolation or compounded with floods, played a crucial role and exerted a profound influence on the total estimated economic costs for Muscat CHN.

**Fig. 6:**
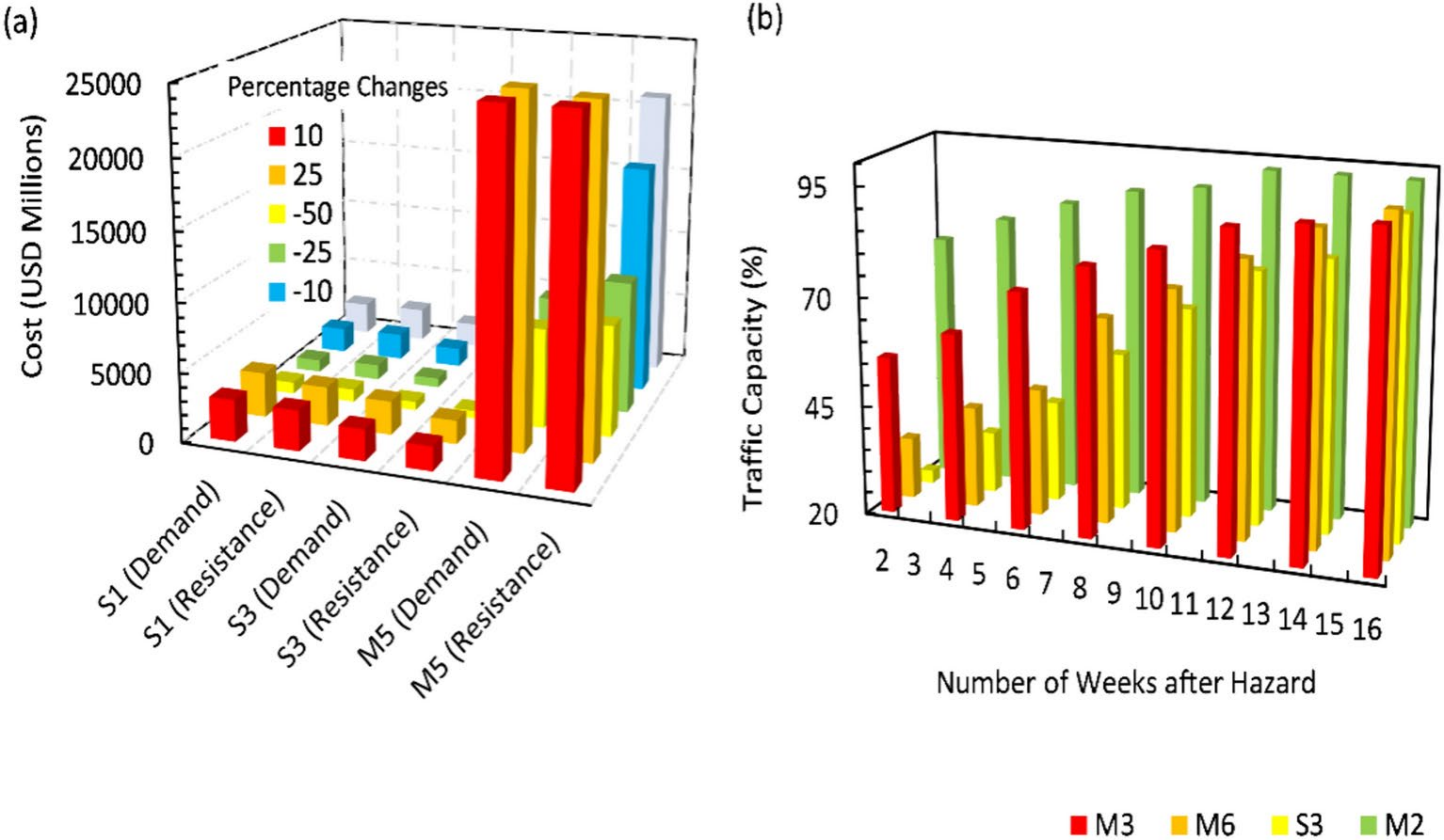
(**a**) Sensitivity of economic costs and (**b**) traffic capacity response over time.

Using Eq. (5), the highway traffic capacity (${C}_{T}$) over a four-month period was estimated, and a sensitivity analysis for economic costs was conducted, as illustrated in Fig. 6. In the S3 scenario, an increase in demand by 25% coupled with a decrease in resistance by 50% led to a substantial rise in economic costs, ranging from 23.54% to 89.42%. This result indicated that the economic impact of flood-induced landslides is highly sensitive to these variations in input parameters. The analysis underscored the significant responsiveness of landslide parameters to changes in median values within the multi-hazard context. In Scenario M5, which includes both seismic-induced liquefaction and flooding, a 36.42% increase in demand resulted in a corresponding rise in cost estimates by 24.32% for flood-related impacts and 17.33% for earthquake-related impacts. This highlighted the critical need to account for multiple interacting factors when evaluating hazard impacts, as minor variations can significantly influence cost estimates. Insights from Fig. 6b revealed that the highway network in scenario M2 experienced relatively rapid recovery, with liquefaction having a lesser impact compared to landslides. Conversely, Scenario S3 demonstrated slower recovery, negatively affecting traffic flow, which suggests that specific hazards in S3 lead to delayed recovery and extended traffic disruptions. These observations emphasized the importance of considering PGD as a key factor in assessing and planning for multi-hazard scenarios.

## Resilience evaluation

**Fig. 7:**
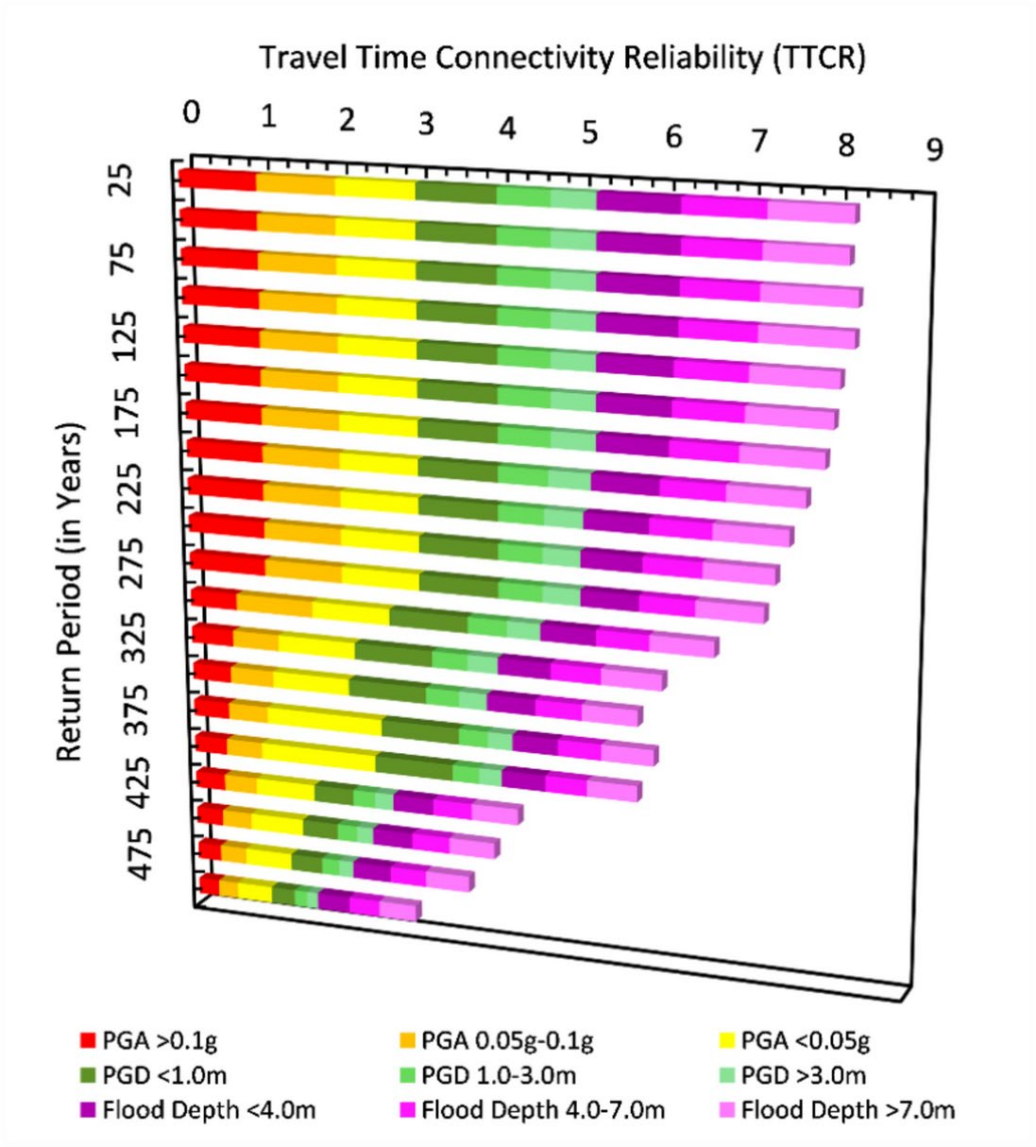
Traffic stability of Muscat CHN for selected range of intensity measure defining earthquake and flood.

The resilience evaluation of Muscat CHN focused on the network ability to endure and recover from multi-hazard scenarios. The traffic capacity was systematically evaluated over four months, allowing for the determination of the Travel Time Connectivity Reliability ($TTCR$) across various RPs (Fig. 7). Notably, the $TTCR$ was relatively stable for earthquake scenarios with RPs less than 275 years, despite increasing traffic demand. However, beyond this threshold, a marked 37.41% decrease in $TTCR$ is observed for each subsequent 25-year RP increment, particularly when PGA and PGD are the dominant intensity parameters. In contrast, the response to flood scenarios revealed a distinct pattern. The $TTCR$ reduction is segmented into three classes based on RP and flood depth. For RPs less than 175 years, and those between 175 to 325 years, the $TTCR$ decreased by 23.45% and 26.87% per 25-year RP interval, respectively. However, for RPs exceeding 325 years, this reduction accelerates to 36.31% for the same increment. These findings highlighted the traffic stability of Muscat CHN under varying RPs for both earthquakes and floods, suggesting that flood-related hazards require significant attention, even at lower RPs, to ensure network resilience. Regarding recovery, the CHN capacity to return to pre-disaster traffic levels depends on the severity and type of hazard.

**Fig. 8:**
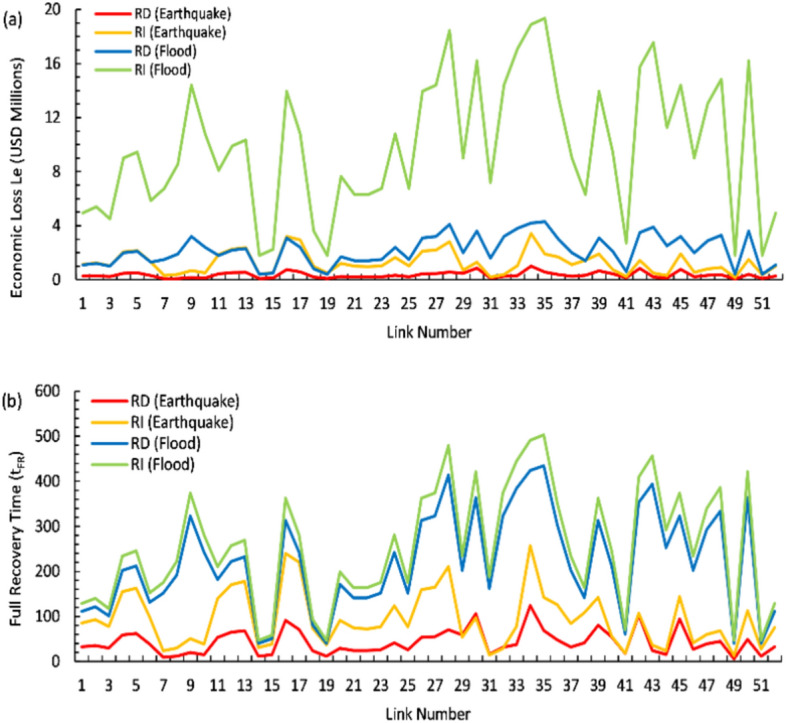
Cost and time analysis of recovery for highway links in multi-hazard scenarios.

Equations (9) through (12) are employed to estimate the economic losses and recovery durations for each link, as illustrated in Fig. 8. Among these links, 18, 6, 24, 28, 34–36, 41–45, and 50 are associated with adjacent bridges that exhibited higher failure probabilities, resulting in elevated economic losses and extended recovery times. Flood-related economic losses are significantly higher than those from earthquakes, with Link 25 experiencing USD 17 million in losses for floods versus USD 3 million for earthquakes. Recovery times for floods also exceed those for earthquakes, as evidenced by Link 30, which takes approximately 280 days for flood recovery compared to 80 days for earthquake recovery. Additionally, links 45 and 50 face increased flood-induced landslide risks and experience higher daily traffic volumes, contributing to greater anticipated economic losses and prolonged recovery periods. Notably, the recovery time is significantly extended for links exceeding 10.2 m in length, suggesting that larger or more critical sections of the highway network are prone to more prolonged disruptions due to these hazards. Comparative analysis indicated that the average economic loss for a given highway link due to flooding is approximately 138.23% higher than that resulting from an earthquake. However, the disparity between direct and indirect losses exhibited a consistent trend across both types of hazards and varying positions of highway links. This suggested a systematic impact of flood versus earthquake events on economic losses, with floods generally resulting in more severe financial consequences for Muscat.

**Fig. 9:**
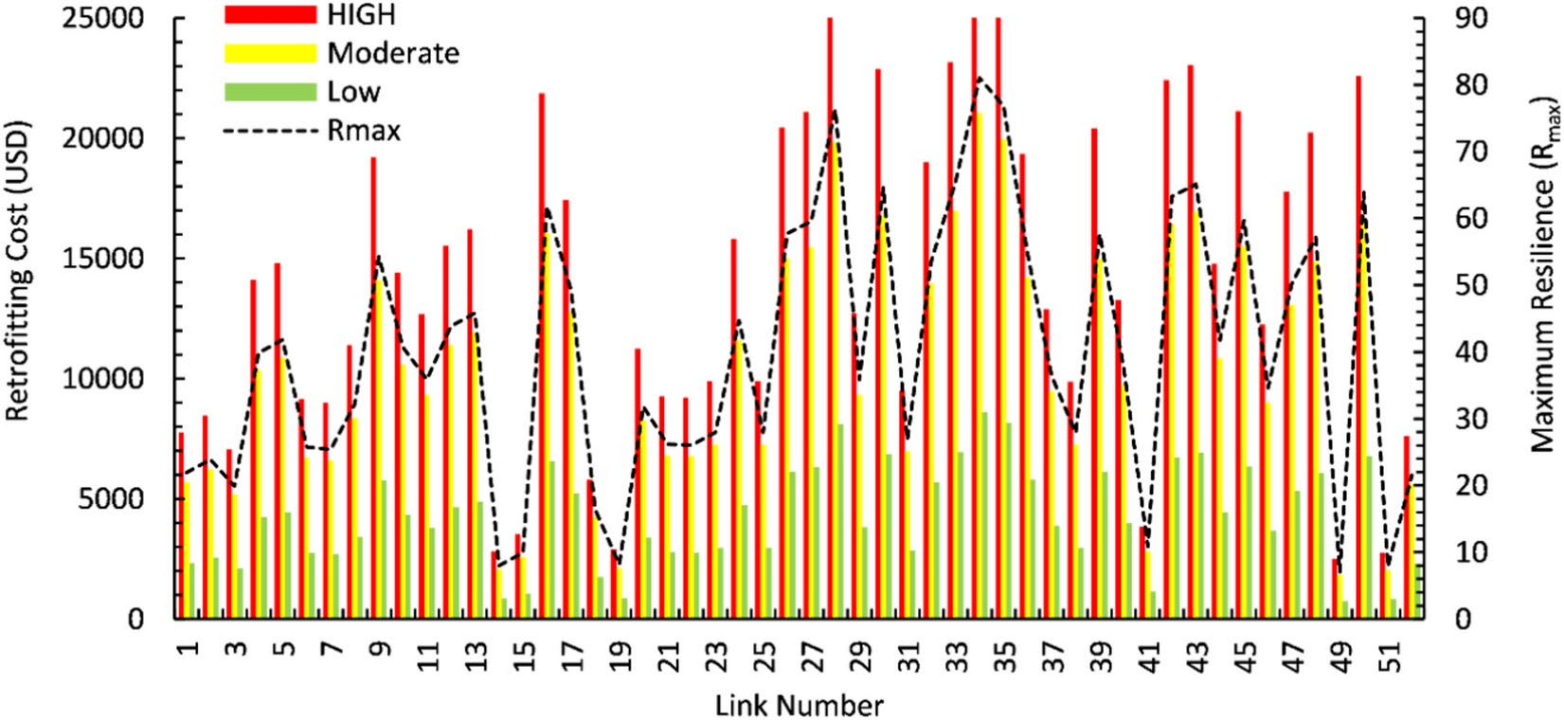
Resilience classes (low, moderate, and high) showing retrofitting costs for each highway link. Black dashed line shows the maximum resilience value.

In this study, Eq. (14) is utilized to calculate the maximum resilience levels for each highway link, which are then categorized into three distinct resilience classes: low, moderate, and high, as illustrated in Fig. 9. The retrofitting costs peak at around USD 25,000 for several links, indicating significant financial investment in these areas, while the resilience values (Rmax) fluctuate, with a maximum observed resilience of approximately 90. Notably, links 28, 34, and 35 demonstrated the highest resilience levels. For flood scenarios, the analysis indicated the following resilience levels in various local areas of Muscat:

1. Low Resilience: Areas such as Al Khuwair and Azaiba are characterized by links with lower resilience levels due to their higher susceptibility to flooding and inadequate infrastructure adaptation.
2. Moderate Resilience: Ruwi and Al Ghubra exhibited moderate resilience, reflecting a balanced capacity to handle flood events with some existing flood management measures.
3. High Resilience: Qurum and Al Mouj showed high resilience, benefiting from robust flood mitigation infrastructure and effective drainage systems.

## Conclusions and future recommendations

This study assessed the robustness of Muscat’s CHN under three secondary hazard scenarios (S1-S3) and six interacting multi-hazard scenarios (M1-M6). Over 50% of highway links require retrofitting, with high resilience observed in Qurum and Al Mouj, while low resilience was identified in Al Khuwair and Azaiba, mainly due to flood susceptibility and infrastructure quality. Aligning with Oman Vision 2040, this research supports resilient and sustainable infrastructure development, contributing to urban modernization, disaster preparedness, economic growth, and long-term infrastructure resilience. Additionally, it advances Oman’s climate change goals by strengthening infrastructure to withstand extreme weather events and reducing vulnerability to climate-induced hazards. Table 5 presents detailed information on low, moderate, and high resilience areas in Muscat, along with their specific adaptation measures.

**Table 5:** Adaptation measures and resilience characteristics for bridges in Muscat.

Resilience zone	Area	% of Bridges by damage class	Link damage index % class	Recovery time class	Retrofitting cost class	Adaptation measure (Flood)	Adaptation measure (Earthquake)
Low resilience	Al Khuwair	Minor: 30%	High (≥ 60%)	Low	High	Construct elevated walkways for pedestrians to ensure safe movement during floods	Strengthen bridge joints with flexible materials to allow for seismic movement
		Moderate: 40%					
		Extensive: 30%					
	Azaiba	Minor: 25%	High (≥ 60%)	Low	High	Create flood retention basins to temporarily store excess runoff	Use seismic isolation bearings on bridges to reduce the transmission of ground motion
		Moderate: 35%					
		Extensive: 40%					
	Bausher	Minor: 35%	High (≥ 60%)	Low	High	Install flood barriers along vulnerable roadways to mitigate flood impact	Enhance the lateral bracing of bridges to improve stability during seismic events
		Moderate: 40%					
		Extensive: 25%					
	Seeb	Minor: 30%	High (≥ 60%)	Low	High	Enhance drainage systems to manage stormwater effectively	Retrofit bridge foundations with deep pilings to resist lateral forces from earthquakes
		Moderate: 50%					
		Extensive: 20%					
Moderate resilience	Ruwi	Minor: 20%	Moderate (30–60%)	Moderate	Moderate	Install permeable pavement on highways to enhance drainage and reduce runoff	Strengthen bridge decks with fiber-reinforced polymers to increase resilience against quakes
		Moderate: 50%					
		Extensive: 30%					
	Al Ghubra	Minor: 15%	Moderate (30–60%)	Moderate	Moderate	Implement green roofs on buildings to absorb rainwater and reduce surface runoff	Install energy-absorbing dampers on bridges to reduce vibrations during seismic events
		Moderate: 55%					
		Extensive: 30%					
	Al Mabelah	Minor: 20%	Moderate (30–60%)	Moderate	Moderate	Adopt rainwater harvesting systems to reduce runoff and manage excess water	Utilize post-tensioning techniques to reinforce bridge components against earthquake forces
		Moderate: 50%					
		Extensive: 30%					
High resilience	Qurum	Minor: 10%	Low (< 30%)	High	Low	Integrate smart drainage systems with sensors for real-time water level monitoring	Design bridges with redundant load paths to maintain structural integrity during seismic events
		Moderate: 40%					
		Extensive: 50%					
	Al Mouj	Minor: 5%	Low (< 30%)	High	Low	Develop natural buffer zones (e.g., wetlands) to absorb floodwaters and protect infrastructure	Reinforce bridge abutments with shear walls to enhance seismic stability
		Moderate: 45%					
		Extensive: 50%					
	Mutrah	Minor: 8%	Low (< 30%)	High	Low	Reinforce coastal barriers to protect against storm surges and flooding	Utilize seismic monitoring systems to evaluate bridge performance during and after seismic events
		Moderate: 42%					
		Extensive: 50%					

Furthermore, it supports United Nations SDG 9 by promoting resilient infrastructure, fostering innovation, and enhancing industrial sustainability through improved highway networks, which in turn ensures stable supply chains and reduces disruptions to critical industries. By minimizing transportation delays and infrastructure damage, the study contributes to efficient industrial operations and sustainable economic growth. This study’s methodology can be applied to various global contexts, including cities like Jakarta, Indonesia, which faces seismic and flooding challenges due to its geographical conditions and rapid urbanization. It can also enhance infrastructure resilience assessments for projects like the California High-Speed Rail. In the Gulf region, cities such as Dubai and Kuwait City can benefit from this framework, addressing flooding and seismic risks. Additionally, the Qatar Integrated Rail Project could utilize this approach to improve infrastructure robustness against multi-hazard scenarios, ultimately supporting sustainable development goals across diverse regions.

## Data Availability

The datasets generated during and/or analyzed during the current study are available from the corresponding author on reasonable request.

## References

[1] Gong, W., Zekkos, D. & Clark, M. The influence of seismic displacement models on spatial prediction of regional earthquake-induced landslides. *Eng. Geol.* **325**, 107288 (2023).

[2] Malik, B. A., Mandhaniya, P., Soni, A. K. & Ouyang, Z. Numerical modeling and failure analysis of caisson foundations subjected to the monotonic lateral loads. *Ocean Eng.* **309**, 118491 (2024).

[3] Mandhaniya, P., Soni, A. K., Choudhary, K. & Ansari, A. A comparison of lateral resistance of geosynthetically reinforced earth foundations for high-speed railways. *Front. Built Environ.* **9**, 1–11. 10.3389/fbuil.2023.1301722 (2023).

[4] Sattar, A., Haritashya, U. K., Kargel, J. S. & Karki, A. Transition of a small Himalayan glacier lake outburst flood to a giant transborder flood and debris flow. *Sci. Rep.* **12**, 12421 (2022). 35858949 10.1038/s41598-022-16337-6 PMC9300610

[5] Wright, S. G. & Rathje, E. M. Triggering mechanisms of slope instability and their relationship to earthquakes and tsunamis. *Pure Appl. Geophys.* **160**, 1865–1877 (2003).

[6] Al-Awadhi, T., Abdullah, M., Al-Ali, Z., Abulibdeh, A., Al-Barwani, M., Al Nasiri, N., & Mohan, M. (2024). Navigating cyclone threats: A forecast approach using water streams’ physical characteristics as an indicator to predict high risk potential areas in the Sultanate of Oman. Earth Syst. Environ., 1–13.

[7] Ansari, A., Lee, J. H., Jang, J. G. & Alluqmani, A. E. Seismic microzonation of North Gyeongsang (South Korea) considering liquefaction potential: Application towards seismic risk assessment for Korean nuclear power plants. *Soil Dyn. Earthq. Eng.* **182**, 108679. 10.1016/j.soildyn.2024.108679 (2024).

[8] Ansari, A., & Rao, K. S. (2024). Evolution of Seismic Fragility Curves (SFC): Configuration and Application for Underground Infrastructure Projects Subjected to Earthquake Hazard. In Geo-Congress 2024 (30–36).

[9] Argyroudis, S. A. & Mitoulis, S. A. Vulnerability of bridges to individual and multiple hazards-floods and earthquakes. *Reliab. Eng. Syst. Saf.* **210**, 107564 (2021).

[10] Argyroudis, S. A. et al. Resilience assessment framework for critical infrastructure in a multi-hazard environment: Case study on transport assets. *Sci. Total Environ.* **714**, 136854 (2020). 32018987 10.1016/j.scitotenv.2020.136854

[11] Elnashai, A. S., Gencturk, B., Kwon, O. S., Al-Qadi, I. L., Hashash, Y., Roesler, J. R., & Valdivia, A. (2010). The Maule (Chile) earthquake of February 27, 2010: Consequence assessment and case studies. MAE Center Report No. 10-04.

[12] Gidaris, I. et al. Multiple-hazard fragility and restoration models of highway bridges for regional risk and resilience assessment in the United States: State-of-the-art review. *J. Struct. Eng.* **143**(3), 04016188 (2017).

[13] Kawashima, K., & Buckle, I. (2013). Structural performance of bridges in the Tohoku-oki earthquake. Earthq. Spectra, 29(1_suppl), 315–338.

[14] Kim, B., Shin, S. C. & Kim, D. Y. Scenario-based economic impact analysis for bridge closures due to flooding: A case study of north gyeongsang province South Korea. *Water* **10**(8), 981 (2018).

[15] Lamb, R., Garside, P., Pant, R. & Hall, J. W. A probabilistic model of the economic risk to Britain’s railway network from bridge scour during floods. *Risk Anal.* **39**(11), 2457–2478 (2019). 31318475 10.1111/risa.13370 PMC6899957

[16] Pucci, A. et al. Fragility analysis based on damaged bridges during the 2021 flood in Germany. *Appl. Sci.* **13**(18), 10454 (2023).

[17] Fu, Z., Gao, R. & Li, Y. Probabilistic seismic resilience-based cost–benefit analysis for bridge retrofit assessment. *Arab. J. Sci. Eng.* **45**, 8457–8474 (2020).

[18] Riedel, I. & Guéguen, P. Modeling of damage-related earthquake losses in a moderate seismic-prone country and cost–benefit evaluation of retrofit investments: Application to France. *Nat. Hazards* **90**, 639–662 (2018).

[19] Tatangelo, M., Audisio, L., D’Amato, M. & Gigliotti, R. Seismic risk analysis on masonry buildings damaged by L’Aquila 2009 and Emilia 2012 earthquakes. *Procedia Struct. Integr.* **44**, 990–997 (2023).

[20] Wang, Q., Liu, K., Wang, M. & Koks, E. E. A river flood and earthquake risk assessment of railway assets along the belt and road. *Int. J. Dis. Risk Sci.* **12**, 553–567 (2021).

[21] D’Amato, M., Laguardia, R., Di Trocchio, G., Coltellacci, M. & Gigliotti, R. Seismic risk assessment for masonry buildings typologies from L’Aquila 2009 earthquake damage data. *J. Earthq. Eng.* **26**(9), 4545–4579 (2022).

[22] Laguardia, R., D’Amato, M., Coltellacci, M., Di Trocchio, G. & Gigliotti, R. Fragility curves and economic loss assessment of RC buildings after L’Aquila 2009 earthquake. *J. Earthq. Eng.* **27**(5), 1126–1150 (2023).

[23] Tatangelo, M., Audisio, L., D’Amato, M. & Gigliotti, R. Issues related to typological fragility curves derivation starting from observed seismic damage. *Eng. Struct.* **307**, 117853 (2024).

[24] Fritz, H. M., Blount, C. D., Albusaidi, F. B. & Al-Harthy, A. H. M. Cyclone Gonu storm surge in Oman. *Estuar. Coast. Shelf Sci.* **86**(1), 102–106 (2010).

[25] Amna, M., Ruheili, A. & Al Wardy, M. The role of geographic information system in environmental planning and management in Oman. *Int. J.* **10**(3), 869–877 (2023).

[26] Aziz, K., Mir, R. A., & Ansari, A. (2024). Precision modeling of slope stability for optimal landslide risk mitigation in Ramban road cut slopes, Jammu and Kashmir (J&K) India. *Model. Earth Syst. Environ.*, 1–17.

[27] Mustafa, A., Szydłowski, M., Veysipanah, M. & Hameed, H. M. GIS-based hydrodynamic modeling for urban flood mitigation in fast-growing regions: A case study of Erbil Kurdistan Region of Iraq. *Sci. Rep.* **13**(1), 8935 (2023). 37264123 10.1038/s41598-023-36138-9 PMC10235075

[28] El-Hussain, I. et al. Seismic microzonation for Muscat region, Sultanate of Oman. *Nat. Hazards* **69**, 1919–1950 (2013).

[29] Ezzelarab, M., El-Hussain, I., Mohamed, A. M. & Deif, A. Site-specific earthquake ground motion parameters at the Southeastern part of Muscat, Sultanate of Oman. *J. Afr. Earth Sci.* **145**, 201–214 (2018).

[30] El-Hussain, I., Saad, M. G., Deif, A., Mohamed, A. E. M. & Ezzelarab, M. Seismic liquefaction potential in Muscat, Sultanate of Oman. *J. Earth Sci. Clim. Change* **9**, 451 (2018).

[31] Sundararajan, N. et al. Shear wave velocity characteristics in parts of Muscat, Sultanate of Oman—a measure of earthquake hazard assessment. *J. Geol. Soc. India* **93**, 515–522 (2019).

[32] Al-Awadhi, T., Charabi, Y., Choudri, B. S. & Bani Oraba, Y. Flooding risk analysis: A case study of Muscat Governorate, Sultanate of Oman. *Hum. Ecol. Risk Assess. Int. J.* **24**(3), 667–678 (2018).

[33] Al-Hinai, H. & Abdalla, R. Mapping coastal flood susceptible areas using shannon’s entropy model: The case of Muscat governorate Oman. *ISPRS Int. J. Geo-Inf.* **10**(4), 252 (2021).

[34] Al Ruheili, A., Dahm, R. & Radke, J. Wadi flood impact assessment of the 2002 cyclonic storm in Dhofar, Oman under present and future sea level conditions. *J. Arid Environ.* **165**, 73–80 (2019).

[35] Hereher, M. et al. Assessment of the coastal vulnerability to sea level rise: Sultanate of Oman. *Environ. Earth Sci.* **79**, 1–12 (2020).

[36] Oman Seismic Design Code for Buildings (OSC). *Earthquake Monitoring Center* (Sultan Qaboos University, 2013).

[37] Ansari, A., Rao, K. S. & Jain, A. K. Seismic response and fragility evaluation of circular tunnels in the Himalayan region: Implications for post-seismic performance of transportation infrastructure projects in Jammu and Kashmir. *Tunnel. Undergr. Space Technol.* **137**, 1–13. 10.1016/j.tust.2023.105118 (2023).

[38] Azad, M. A. et al. Development of correlations between various engineering rockmass classification systems using railway tunnel data in Garhwal Himalaya India. *Sci. Rep.* **14**(1), 10716 (2024). 38729957 10.1038/s41598-024-60289-y PMC11087513

[39] F. E. M. A. (2010). HAZUS-MH MR5 technical manual—Earthquake model. Washington, DC: FEMA.

[40] Nako, A., Shlke, C., Six, J., & Johnsom, B. (2009). Seismic vulnerability of Oregon state highway bridges: Mitigation strategies to reduce major mobility risks (No. OR-RD-10-08). Oregon. Dept. of Transportation.

[41] Mehary, S. T., & Dusicka, P. (2015). Seismic retrofit benefit considering statewide transportation assessment. Report OR-RD-15-15. Portland, OR: Transportation Research and Education Center (TREC), 2015. 10.15760/trec.123

[42] Chang, S. E., Shinozuka, M. & Moore, J. E. Probabilistic earthquake scenarios: Extending risk analysis methodologies to spatially distributed systems. *Earthq. Spectra* **16**(3), 557–572 (2000).

[43] Dong, Y. & Frangopol, D. M. Risk and resilience assessment of bridges under mainshock and aftershocks incorporating uncertainties. *Eng. Struct.* **83**, 198–208 (2015).

[44] Fan, X., Zhang, X., Wang, X. & Yu, X. A deep reinforcement learning model for resilient road network recovery under earthquake or flooding hazards. *J. Infrastruct. Preserv. Resil.* **4**(1), 8 (2023).

[45] Lee, J. H., Ansari, A., An, H. & Jeong, J. Y. Seismic loss and resilience modelling of bridges in soft soils: Towards design of sustainable transportation infrastructure facilities. *Sustain. Resil. Infrastr.* **9**(2), 1–23. 10.1080/23789689.2024.2328979 (2024).

[46] Ishibashi, H. et al. Framework for estimating the risk and resilience of road networks with bridges and embankments under both seismic and tsunami hazards. *Struct. Infrastr. Eng.* **17**(4), 494–514 (2020).

